# A Case of Sickle Cell Retinopathy With Retinal Artery Occlusion in African-Japanese Patients

**DOI:** 10.7759/cureus.60653

**Published:** 2024-05-20

**Authors:** Yoshiki Kato, Taro Kominami

**Affiliations:** 1 Ophthalmology, Japanese Red Cross Aichi Medical Center Nagoya Daiichi Hospital, Nagoya, JPN; 2 Ophthalmology, Nagoya University, Nagoya, JPN

**Keywords:** japanese, ophthalmology, retinal artery occulusion, sickle cell retinopathy, sickle cell disease

## Abstract

As globalization progresses, cases of sickle cell disease (SCD) are now being seen even in Japan, where SCD did not originally exist. SCD causes not only anemia but also peripheral blood flow obstruction, which can lead to systemic complications. This report represents a case of sickle cell retinopathy (SCR) in Japan discovered with the onset of retinal artery occlusion (RAO). The patient, a 20-year-old African-Japanese male, was being monitored for SCD at the Nagoya University Hospital, Pediatrics Department, Nagoya, Japan. Following a chest pain episode, he reported a loss of vision in his right eye and was referred to the ophthalmology department. Examination showed reduced visual acuity in the right eye 20/40 compared to the left 20/20. A Goldman visual field test indicated central vision loss in the right eye, and fundoscopic examination revealed yellow-white lesions centered on the macula and peripheral salmon-patch-like lesions in the right eye, with peripheral black sunburst-like lesions in the left eye. Optical coherence tomography (OCT) of the right eye showed inner retinal edema within the macula, suggesting an SCR accompanied by branch RAO. Six months later, he complained of further vision loss in his right eye. Examination and OCT revealed sub-inner limiting membrane hemorrhage in the right eye, suggesting worsening of the SCR. SCD is exceedingly rare among native Japanese but is likely to be encountered more frequently as globalization progresses. Even in countries where SCD has traditionally been rare, attention must be paid to the occurrence of severe SCR when managing SCD.

## Introduction

Sickle cell retinopathy (SCR) represents a consequential complication of sickle cell disease (SCD), a genetic disorder [1] predominantly affecting individuals of African descent [2,3], but increasingly observed globally due to population migration [4-6]. Characterized by the sickling of red blood cells under low oxygen tension, SCD disrupts normal blood flow, precipitating a spectrum of ocular manifestations, including proliferative retinopathy and retinal artery occlusion [7].

Despite extensive documentation of SCR in the European or American population, the presentation within the East Asian population remains poorly characterized. The SCR's clinical course in the East Asian population may not be widely known to ophthalmologists in this region, particularly concerning the onset and severity of complications such as retinal artery occlusion and its management. Here, we present an African-Japanese SCR case with retinal artery occlusion.

## Case presentation

We document the case of a 20-year-old African-Japanese male affected with SCR. This patient was under long-term pediatric observation at Nagoya University Hospital for SCD. Genetic testing was not performed, but blood tests confirmed the presence of sickle cell. This patient presented with a sudden episode of chest pain, followed by a unilateral visual field loss in his right eye. He was referred to the Department of Ophthalmology, and ophthalmic observation revealed that reduced best corrected visual acuity (BCVA), measuring 20/40 in the right eye, significantly diminished from the baseline measurement of 20/20. His left eye maintained a BCVA of 20/20. Intraocular pressures were within normal limits at 10 mmHg for the right eye and 14 mmHg for the left. The anterior segment of the eye displayed no remarkable findings.

**Figure 1:**
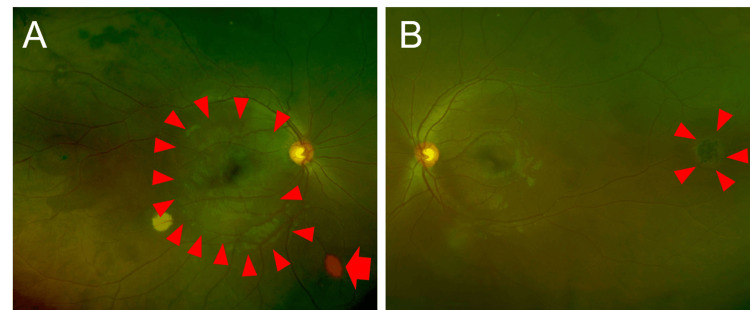
Wide-field fundus photographs Wide-field fundus photographs (A: right eye, B: left eye). The area surrounded by the arrowhead in Figure 1A shows a yellowish-white change, suggesting retinal edema. The arrow in Figure 1A shows the sign of ‘salmon patch’. In Figure 1B, there was a ‘black sunburst’ sign due to peripheral RPE hyperplasia on the temporal side of the fundus of the left eye. RPE, Retinal pigment epithelium

**Figure 2:**
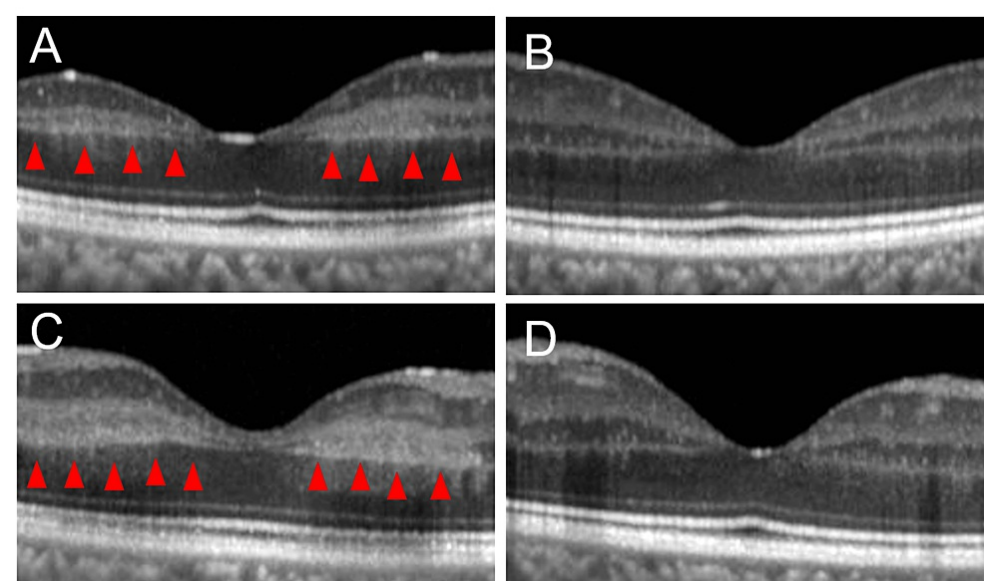
OCT images Images of horizontal retinal sections around the fovea were shown in Figures 1A (right eye) and 1B (left eye), and images of vertical retinal sections around the fovea were shown in Figures 1C (right eye) and 1D (left eye). Arrowheads show inner retinal edema. OCT, Optical coherence tomography

Detailed fundoscopic examination revealed distinct yellow-white macular lesions (Figure 1A) and peripheral retinal changes characteristic of sickle cell retinopathy-salmon patches (Figure 1A) and black sunbursts (Figure 1B), which are indicative of localized retinal hemorrhage and reactive pigmentation respectively. Optical coherence tomography (OCT) also showed inner retinal edema in the macula (Figures 2A, 2C).

Diagnostically, these findings led to the suspicion of a branch retinal artery occlusion secondary to SCR, a rare but severe manifestation of SCD affecting the ocular vasculature. The fluorescein angiography or OCT angiography was not tested to confirm the occlusion of the retinal artery, and the interventional approaches were not chosen because the ocular complications were not acute, with a few days between onset and consultation.

Over the course of his treatment, there were no invasive procedures undertaken immediately; however, regular follow-ups were scheduled to monitor the progression or resolution of the ocular findings and to adjust care plans as necessary based on the dynamic nature of his disease presentation.

## Discussion

In the hemoglobinopathies manifesting in ocular tissues, our case highlights two pivotal findings that extend the discourse on SCR within a Japanese population, which has been traditionally perceived as low risk due to its homogeneity in Japan. Firstly, the rarity of SCR in Japan is historically attributed to the ethnic and genetic uniformity of the population. However, with globalization, the ethnic mosaic of Japan is becoming increasingly variable, presenting new clinical challenges in the diagnosis of diseases once considered unfamiliar in Japanese due to its ethnic unity. This demographic shift warns Japanese medical doctors to be careful about diagnostic acuity for SCR. Secondly, the pathophysiology of SCR, likely accompanied by abnormalities in the blood coagulation system, imposes the potential for acute ophthalmological emergencies, such as retinal artery occlusion. This emergent risk profile calls for rigorous, proactive ophthalmologic surveillance to prevent and reduce the severe aftereffects of this condition.

Our case report may reflect an emerging trend in the epidemiology of SCR in Japan, mirroring the global shift towards increased ethnic diversity. Traditionally, SCR has been considered exceptionally rare in Japan [8], largely due to the genetic homogeneity that characterizes the Japanese population not having SCD originally and the low immigration to Japan from other countries so far. It is estimated that hemoglobinopathy affects approximately one in 3,000 people in Japan. However, SCD is extremely rare, and, to the best of the authors' knowledge, the annual number of diagnoses of SCD cases in Japan has not been reported. However, our case report illustrates that the evolving demographic landscape due to globalization is likely to increase the incidence of SCR in Japan. This assertion aligns with the previous study on the impact of global migration on the distribution of the sickle-cell gene, finding a substantial effect due to increased international migrations from countries with high hemoglobin S allele frequencies [9]. Highlighting our first finding once more, it is important to acknowledge the increasing likelihood of encountering SCR in Japan, necessitating greater awareness and diagnostic capabilities among healthcare providers.

Concerning our second finding, the pathophysiological mechanism underlying SCR, primarily driven by abnormalities in the blood coagulation system, suggests significant risks for ophthalmologic emergencies such as retinal artery occlusion. This complication has been considered to be caused by the unique rheological properties of sickled red blood cells, which promote occlusion and ischemic damage [10]. On the contrary, there were several studies that discuss various aspects of SCD that imply a more complex interaction with coagulation processes than just promoting arterial occlusions such as deoxygenation and vascular inflammation leading to hemoglobin polymerization, which increases blood viscosity and reduces flow velocity [11,12].

The implications of these findings are various. Firstly, they impose the necessity of integrating genetic and demographic trends into public health surveillance and medical education in Japan. By anticipating changes in disease patterns associated with population shifts, healthcare systems can better prepare for emerging challenges. Secondly, the recognition of SCR’s serious complications informs clinical practice, emphasizing the importance of early and aggressive management strategies to reduce the risk of vision loss. Furthermore, this study highlights the critical need for interdisciplinary collaboration, particularly involving hematologists and ophthalmologists, to enhance patient outcomes through comprehensive care approaches.

## Conclusions

In conclusion, our study not only illuminates the shifting epidemiology of sickle cell retinopathy in a traditionally low-incidence region but also emphasizes the critical nature of this condition due to its association with severe ocular complications. The findings from this case are important for clinical practice, advocating for heightened surveillance, proactive management, and tailored patient education to address and reduce the risks associated with SCR. As Japan's demographic profile continues to diversify, the insights obtained here will undoubtedly enhance the preparedness and responsiveness of its healthcare system to effectively manage and treat SCR.
